# Multimodal tilmanocept for preoperative imaging and fluorescence-guided surgery of porcine lateral pelvic sentinel lymph nodes

**DOI:** 10.1038/s41598-025-21160-w

**Published:** 2025-10-27

**Authors:** Ryotaro Ogawa, Junichiro Kawamura, Edward T. Ashworth, Soo Bin Park, David J. Hall, Masatoshi Kudo, Carl K. Hoh, David R. Vera

**Affiliations:** 1 Moores Cancer Center, University of California, San Diego, La Jolla, CA USA; 2 Department of Surgery, Kindai University Faculty of Medicine, Osakasayama, Osaka Japan; 3 Department of Radiology, University of California, San Diego, La Jolla, CA USA; 4 Department of Gastroenterology and Hepatology, Kindai University Faculty of Medicine, Osakasayama, Osaka Japan

**Keywords:** Rectal cancer, Lateral pelvic lymph node, Sentinel lymph node, Tilmanocept, Robotic surgery, Fluorescent navigation surgery, Biological techniques, Cancer

## Abstract

The management of lateral pelvic lymph nodes in locally advanced lower rectal cancer remains controversial. The sentinel lymph node (SLN) mapping can potentially enhance patient selection for lateral pelvic lymph node dissection. The current mapping agent, indocyanine green dye cannot be externally imaged prior to surgery and is not retained after entering the SLN. This study evaluated the ability of tilmanocept, a receptor-specific SLN mapping agent, to provide preoperative PET cross-sectional imaging and sustained intraoperative fluorescence images during rectal cancer surgery. Tilmanocept was labeled with gallium-68, technetium-99m, and a near-infrared fluorophore. Four pigs were studied. Tilmanocept was injected into the submucosal layer of the rectal wall followed 1 h later by PET/CT images of the pelvis, which identified ten SLNs, one of which was a pudendal artery regional SLN. Approximately 45 h after administration, SLN dissection was guided by fluorescence imaging during robotic surgery. The tenth SLN was excised by open surgery. The radioactive and fluorescent intensities of all ten excised SLNs were significantly higher than the non-SLNs. The ability of dual-labeled tilmanocept to provide preoperative SLN imaging can potentially reduce morbidity and operation time by identifying patients who will not require lateral pelvic lymph node dissections.

## Introduction

Increasing attention has been focused on treatment strategies for lateral pelvic lymph nodes (LPLN) in locally advanced lower rectal cancer. Total mesorectal excision (TME) is the standard procedure in rectal cancer surgery worldwide. TME has been shown to control local recurrence and improve survival^1^. Recurrence in the lateral pelvic lymph nodes, which are not included in the TME procedure, remains an issue. Treatment of the lateral pelvic lymph nodes is essential to control local recurrence^2^ and improve survival in patients with rectal cancer. Treatment strategies for locally advanced lower rectal cancer differ between Western countries and Japan, and a consensus has not yet been reached. Chemoradiation therapy (CRT) and TME are used in Europe and the United States^3,4^. In Japan, lateral pelvic lymph node dissection (LPLND) has been widely used as a standard procedure in addition to TME^5^. In some patients at risk of LPLN disease, both treatments may be inadequate for the lateral pelvic lymph nodes although CRT or LPLND provide better local control than TME alone^3^. The treatment strategy is shifting to CRT with TME and LPLND for high-risk LPLN-positive cases^6^.

In recent years, preoperative treatments such as total neoadjuvant therapy have become more common worldwide, and the number of CRT-treated cases is also increasing in Japan. In some patients with enlarged clinical metastatic-positive lateral pelvic lymph nodes, CRT alone may be insufficient treatment to eradicate metastasis of lateral pelvic lymph nodes^7–9^, necessitating the addition of LPLND. However, LPLND is a complex procedure that requires preserving the autonomic nervous system to prevent sexual dysfunction and dysuria, significantly prolonging operative time and increasing blood loss^10,11^. Therefore, it is crucial to identify eligible patients so that LPLND does not become overtreatment. In general, size cut off criteria such as 5–10 mm are commonly used for the preoperative evaluation of lateral pelvic lymph nodes^9,12,13^ although size, shape, intensity and FDG-PET uptake are considered^14^. However, there are no gold standard criteria for preoperative evaluation of metastatic lymph nodes. To give the best possible patient care, there is a clinical need to develop a new, more reliable diagnostic method.

Today, the sentinel lymph node concept is clinically applied in breast cancer and melanoma using Tc-99m-tilmanocept, various radiolabeled colloids, and dyes^15–18^. SPECT/CT scans of Tc-99m-tilmanocept enable cross-sectional imaging for SLN mapping of head and neck cancer^19^. In colorectal cancers, navigation surgery in which ICG is administered near the tumor to visualize the lymphatic flow and the dominant lymph nodes is also performed^20–22^. Tc-99m-tilmanocept was approved by the United States FDA for sentinel lymph node mapping of solid tumors in 2013.

Fluorescent-labeled tilmanocept^23^ and radiolabeled-tilmanocept^24^ exhibit sub-nanomolar affinity for CD206, a surface receptor of macrophages^25^ and dendritic cells^26^. We have reported the use of multimodal tilmanocept to identify sentinel nodes in animal models of the prostate^27^, bladder wall^28^, endometrium^29^, and oral cavity^30^. A gallium-68 label, the radioactive mode, enabled pre-operative imaging with a PET/CT scanner, and a near-infrared label, the fluorescence mode, enabled intra-operative imaging during robotic surgery.

When the SLN concept is applied to rectal cancer with complex lymphatic-systems, it may help selecting patients for LPLND^31^ and avoid unnecessary LPLND. Fluorescent-guided surgery with preoperative imaging could substantially reduce morbidity and operation time. In this study, we investigated whether a CD206-specific multimodal tilmanocept could identify sentinel lymph node locations using PET/CT preoperative imaging and intraoperatively under fluorescence guidance using the *Firefly* (Intuitive, Sunnyvale, CA) camera system.

## Methods

### Experimental design

We designed a non-survival study using four female Yorkshire pigs (Premier BioSource, Ramona CA) to evaluate a clinical protocol for sentinel lymph node mapping of patients with rectal cancer. The animals ranged in age from 85 to 90 days and weighed between 31 and 32 kg. The primary goal of this study was a demonstration of bi-modal tilmanocept for pre-operative cross-sectional imaging and intra-operative sentinel lymph node mapping many days after administration. We labeled tilmanocept with a near-infrared fluorescent dye (*IRDye800CW*) and two radioisotopes: gallium-68, and technetium-99m. Gallium-68 is a positron emission isotope with a 68-min half-life, which enabled pre-operative PET imaging of tilmanocept for in vivo sentinel lymph node mapping and surgical planning. Technetium-99m is a gamma-emitting isotope, with a 6-h half-life, which provided a measurement of lymph node accumulation, and therefore, an independent identification of SLN status. A radiologist or nuclear medicine physician examined the resulting attenuation-corrected cross-sectional images, identified sentinel lymph nodes based on SUVs and the 10%-rule, and preoperatively informed the surgeon as to the anatomic location.

### Animal model and reagents

The protocol for animal transfer and experiments was approved by the University of California, San Diego Institutional Animal Care and Use Committee. All animal procedures were performed in accordance with (1) the Guide for the Care and Use of Laboratory Animals as published by the National Research Council, (2) Association for Assessment and Accreditation of Laboratory Animal Care regulations, and (3) ARRIVE guidelines. A total of four pigs female were studied. During transportation to the imaging or surgical facility, each pig was sedated by intramuscular injection of an acepromazine/buprenorphine (0.10–0.05 and 0.01 mg/kg) cocktail. All procedures were performed under general anesthesia (propofol i.v., 2 mg induction, 10 mg/kg/h maintenance). No animals were excluded from the study.

Tilmanocept was obtained from Navidea Biopharmaceuticals (Dublin, OH), and *IRDye800CW*-NHS-ester was purchased from LICOR Biosciences (Lincoln, NB). Fluorescent-labeled tilmanocept was prepared by covalent attachment of *IRDye800CW* to tilmanocept using a previously described method^32^. *IRDye800CW*-tilmanocept (1.5 dyes per tilmanocept) was radiolabeled with technetium-99m and gallium-68 as previously reported^33^. The radiochemical yield (RCY) and fluorescent purity were measured by ITLC using *Whatman31* as the stationary phase and acetone as the mobile phase. The RCYs and fluorescent purity of all preparations exceeded over 98%.

### PET/CT imaging

The pigs were positioned (right lateral recumbent) within the gantry of a *uMI550* PET-CT scanner (United Imaging, Houston TX) so that the injection of 0.1 ml of ^68^Ga-^99m^Tc-labeled *IRDye800CW*-tilmaoncept (1.5 nmol Tilmanocept, ~ 5 MBq ^68^Ga, 30 MBq ^99m^Tc) could be performed above the dentate line on the rectal side wall. Scout CT images were taken for correct positioning followed by a CT scan for attenuation correction. Whole-body PET-CT images were acquired at approximately 60 min post-injection and consisted of two bed positions spanning from the rectum to the lower chest of each animal. Each bed position was acquired for 2 min. After recovery from anesthesia, each pig was returned to the vivarium.

The PET images were reconstructed with CT attenuation correction using the scanner’s software. The hardware-fused PET and CT cross-sectional images (2.85 mm thickness) were reviewed and PET standard uptake values (SUV) measurements were made with a medical imaging viewer (*Visage 7* Visage Imaging; Irvine, CA). If multiple lymph nodes were visualized, the lymph node with the highest SUV was declared a sentinel lymph node; any of the remaining lymph nodes qualified as a SLN, if it had an SUV greater than 10% percent of the first SLN. In addition to calculating SUVs, the Visage software was used to measure distances from anatomical landmarks. This was accomplished by selecting the Distance Measurement tool from *Visage 7*, which permitted the selection of two measurement points within an image. Based on the pixel spacing value within the image’s DICOM header, Visage computed the Euclidean distance between the points and displayed the distance within the image in millimeters. Prior to each robotic-assisted surgery, the surgeon was provided with the fused PET/CT images of each sentinel lymph node. This information typically consisted of fused PET/CT cross-section of at least two orientations (coronal, transaxial, and/or sagittal), allowing a measured distant from an anatomical landmark, typically a boney structure delineated from the fused PET/CT cross-section.

### Robotic-assisted surgery and *Firefly* imaging

Within 2 days of PET/CT imaging, each pig was transferred to the UCSD Center for the Future of Surgery, where each animal was instrumented with a *da Vinci* surgical system (*Model Xi*, Intuitive Inc; Sunnyvale CA). The pigs were anesthetized and set on the bed in the supine position. A camera port was placed on the midline, one on the left abdomen, and two on the right. The uterus was hung to the abdominal wall, and lymph node dissection started. Lymph nodes around the external and internal iliac arteries were dissected from the internal and external iliac artery bifurcation toward the anal side. The lateral pelvic lymph nodes of both sides were excised completely. In addition, paraaortic lymph node near inferior mesenteric artery and pudendal artery regional lymph node were sampled. Finally, the mesorectum was dissected, and the mesorectum lymph nodes were excised from around the rectum. Guided by the preoperative PET/CT images, the *Firefly* camera system (Intuitive, Inc; Sunnyvale CA) was used to localize the fluorescence of each lymph node during the resection. Both *Firefly* and *sensitive* modes were employed.

### Radioactivity and fluorescence measurements

The dissected lymph nodes were individually separated from the bulk of lymph nodes embedded in fat tissue. Each lymph node was placed in a plastic scintillation vial and measured for technetium-99m radioactivity (100–200 keV energy window) in an autowell gamma counter (*Wizard2*, PerkinElmer, Waltham, MA) with a counting standard, which consisted of a known amount of injectate of a known dilution. The percent-of-injected dose (*%ID*) was calculated using the counting standard. All lymph nodes were imaged with a hand-held fluorescence imaging system (*Fluobeam800*, Fluoptics, Grenoble, France). Separated lymph nodes images were acquired with a 10-ms exposure time. The fluorescence images were analyzed using *ImageJ* (NIH, Bethesda MD); ROIs were drawn around each lymph node and the maximum gray luminance value was obtained within each ROI. We followed the “10% rule” to identify sentinel lymph nodes^34^. According to this rule, lymph nodes that accumulated more than 10% of the highest radioactivity in technetium-99m measurement qualified as sentinel lymph nodes.

### Statistical analysis

Statistical analysis was performed with JMP® *Pro 16.0.0*. Percent-of-injected dose and fluorescent intensity were assessed by the two-tailed Student’s t-test. We considered a *P* value less than 0.05 to be statistically significant.

## Results

### Preoperative PET/CT imaging

**Fig. 1:**
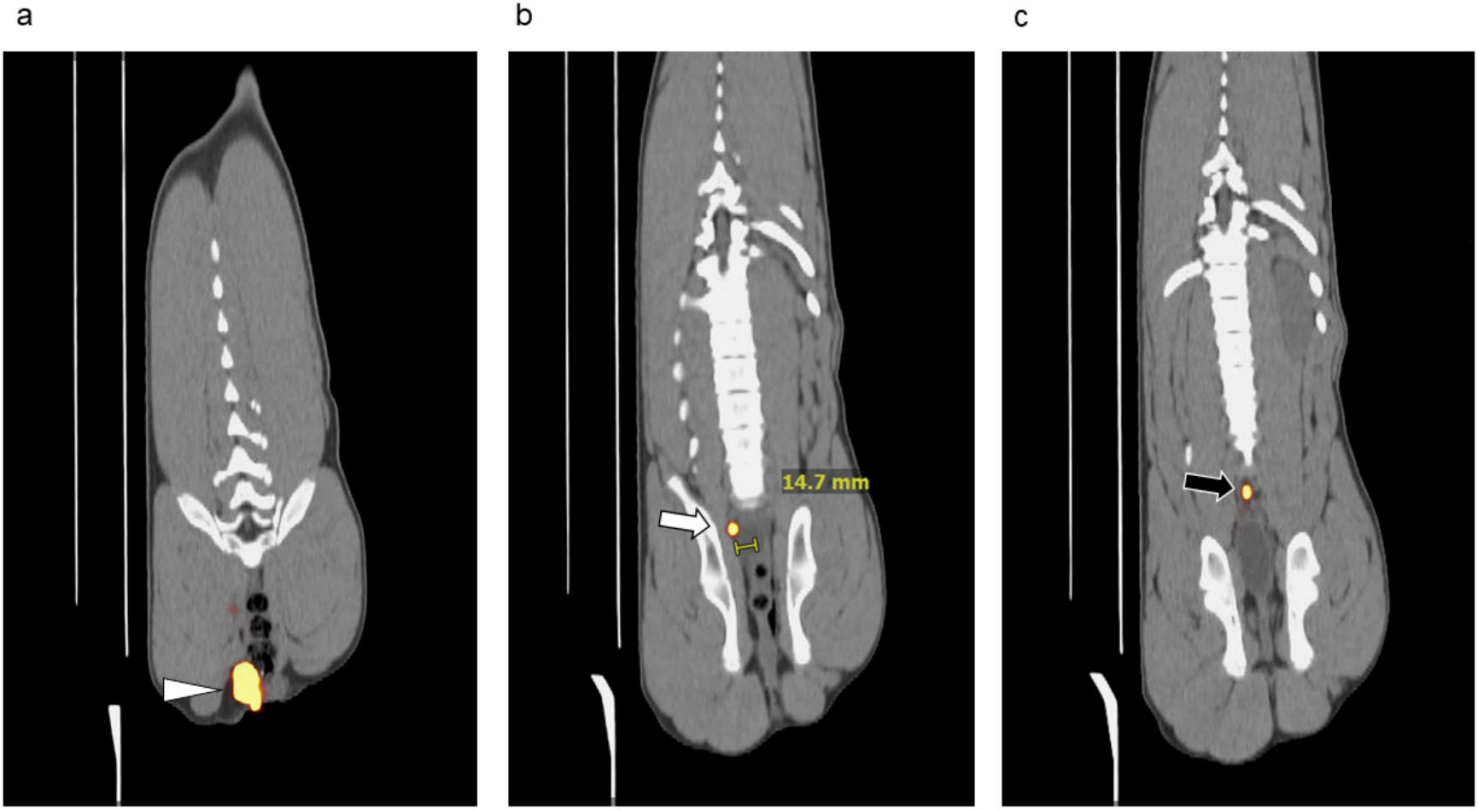
Preoperative coronal PET/CT images of Pig#3 at 1 h after tilmanocept injection. Gallium-68-labeled Tilmanocept was injected into the lateral wall just above the dentate line. The pig was in the right lateral recumbent position. Tilmanocept accumulated in the ipsilateral lymph node of the injection site. (**a**) Right-side injection site (arrowhead) (**b**) right internal iliac regional lymph node, 14.7 mm from midline, SUVmax = 1902 (white arrow). (**c**) Right internal iliac regional lymph node, at midline, SUVmax = 835 (black arrow). The cross-section of (**c**) is dorsal to the cross-section of (**b**) by 5.7 mm.

Preoperative PET/CT imaging identified a total of 10 lymph nodes from four pigs. The mean number of lymph nodes was 2.5 (range 1–4). The maximum standardized uptake value (SUVmax) was 95–1902 (Table 1) and that of injection site was 51,470–93,275. PET/CT detected lymph nodes in intra-mesenteric or ipsilateral lateral pelvic regions (see Table 1). Two of four pigs showed mesenteric and lateral pelvic lymph nodes due to both vertical and parallel lymph flow, and in some cases, images of lymphatic flow migrating through the lymphatic vessels could be visualized. The three panels of Fig. 1 are representative PET/CT cross-sections from Pig#3. The injection site is shown in Fig. 1a. Two ipsilateral sentinel lymph nodes (Fig. 1b,c) along the right internal iliac artery had SUVmax values of 1902 and 835, respectively. The SLN of Fig. 1b was 14.7 mm for the midline and the SLN of Fig. 1c was estimated to be on the midline. The cross-sections of Fig. 1b and c are separated by a single 2.85 mm cross-section; therefore, the SLN of Fig. 1c is dorsal to the cross-section of Fig. 1b by 5.7 mm.

**Table 1:** Comparison of Tilmanocept accumulation in sentinel lymph nodes and visualization by fluorescence

Case #	Injection site	SLN location	PET/CT SUV	*Da Vinci Firefly*	Fluobeam max (IU)	Tc-99m %ID (%)
1	Left	Lt. external Iliac	104	Y	4848	1.73
		Lt. internal Iliac	535	Y	4064	1.99
		Mesenteric	409/396*	Y	3232	0.85
		Mesenteric		Y	2976	0.42
2	Left	Lt. internal Iliac	95	Y	9840	2.25
3	Right	Rt. internal Iliac	835	Y	11,200	2.80
		Rt. internal Iliac	1902	Y	9120	1.49
4	Right	Rt. pudendal	419	**	6016	4.19
		Mesenteric	318*	Y	4384	1.81
		Mesenteric		Y	3952	1.43

*Indistinguishable.

**Out of *Firefly* detection range.

**Fig. 2:**
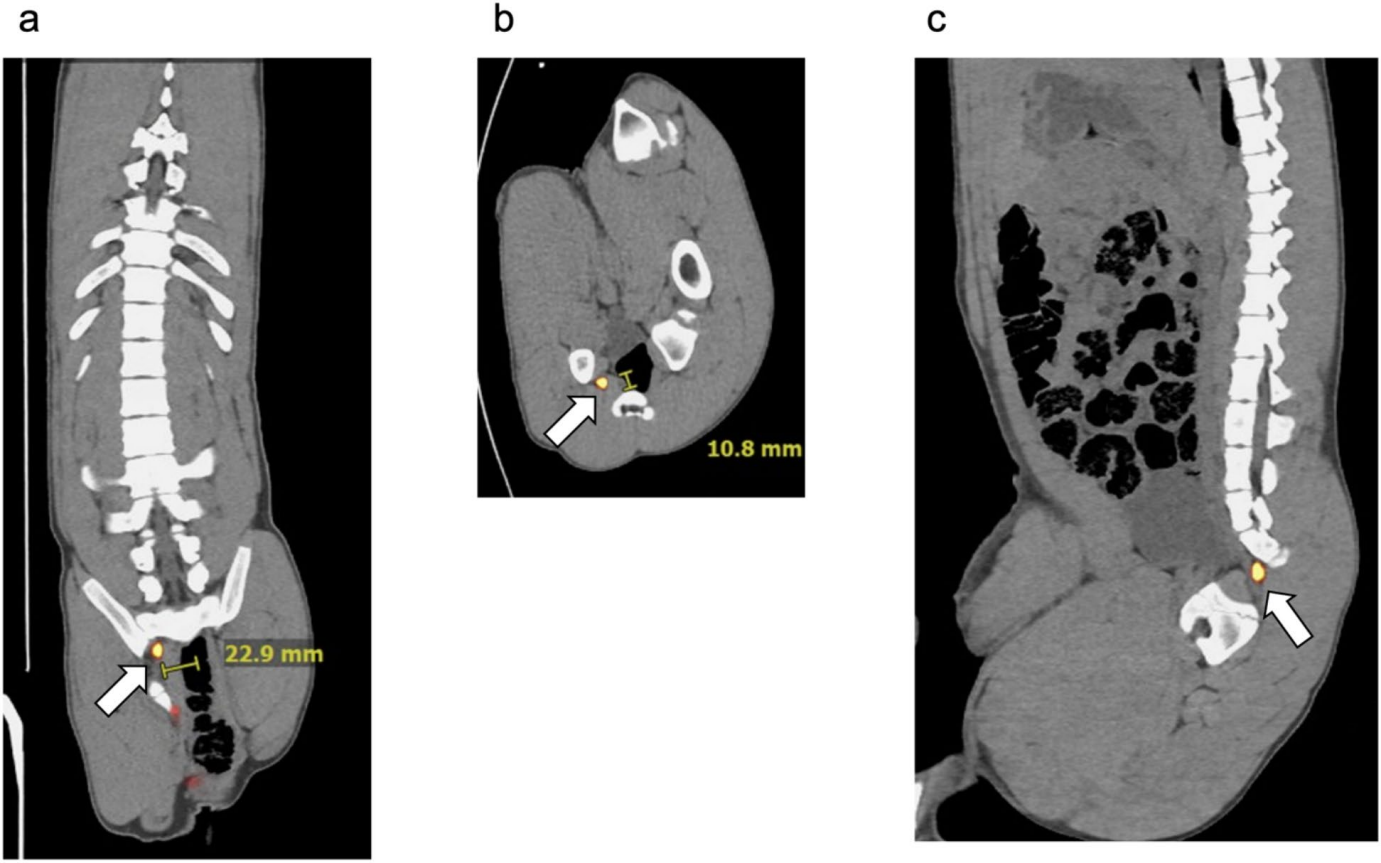
Preoperative PET/CT images of Pig#4 at 1 h after tilmanocept injection. Gallium-68-labeled Tilmanocept was injected into the lateral wall just above the dentate line. The pig was in the right lateral recumbent position. Tilmanocept accumulated in the right pudendal artery regional lymph node (arrow): (**a**) coronal view (SUV = 419), and (**b**) transaxial view, (**c**) sagittal view. Note short distance to pelvic bone and measurements from midline (22.9 mm) and sacrum (10.8 mm) which could be used to assist in intraoperative sentinel node localization. The two red foci, one at the tip of a pelvic bone and other at the rectum, are the result of radiation photon scatter from the injection site activity.

Pre-operative PET/CT imaging of Pig #4 showed a right pudendal artery regional sentinel lymph node (arrow); coronal (SUV = 419), transaxial, and sagittal cross-sections are displayed in Fig. 2a–c, respectively. Note the measured distances of the sentinel lymph node provided by the viewing software: 22.9 mm from anatomic midline in the coronal view and 10.8 mm from the sacrum in the transaxial view.

### Intraoperative *Firefly* fluorescence imaging identifies sentinel lymph nodes

**Fig. 3:**
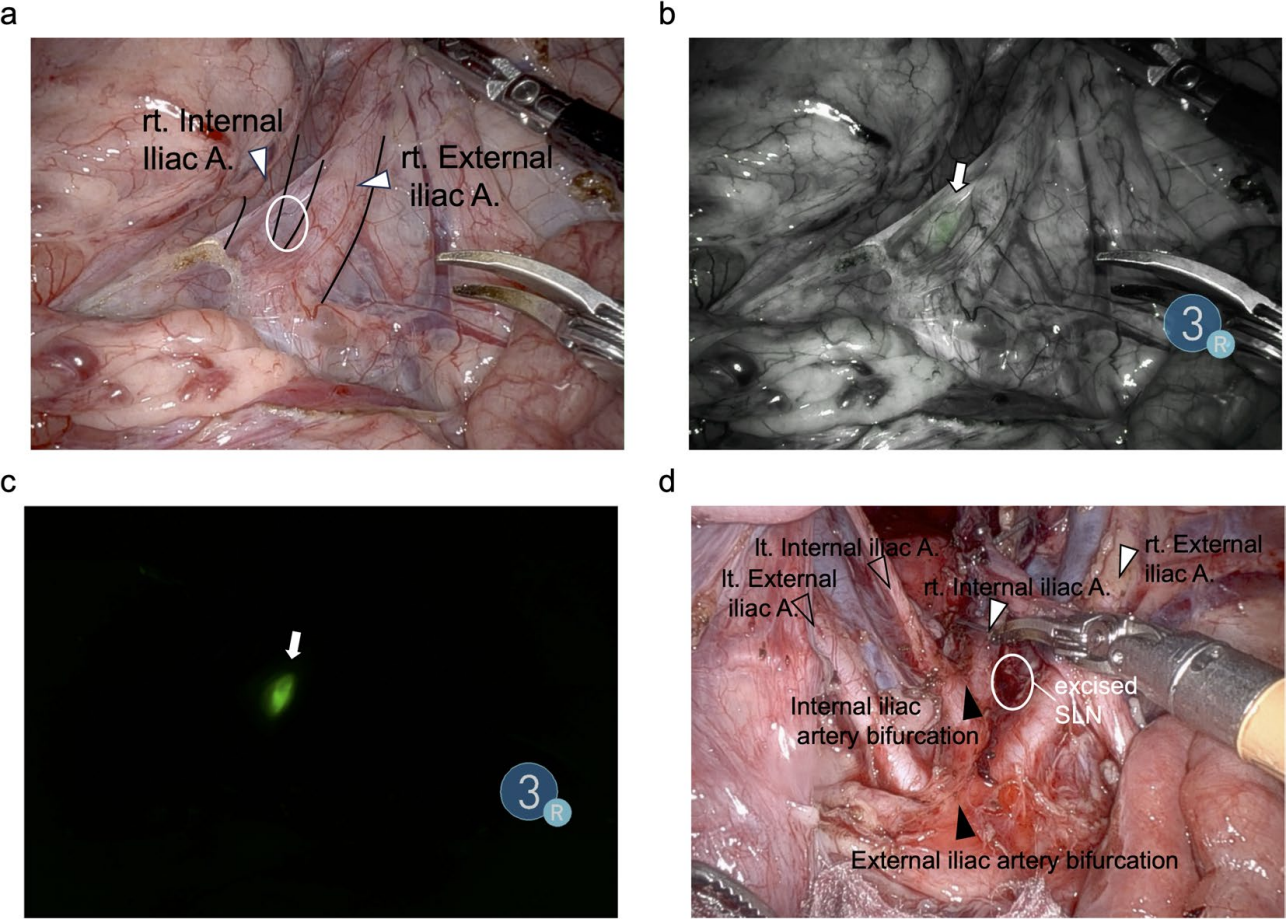
Intraoperative Pig#3 images at the midline were acquired approximately 45 h after injection of *IRDye800CW*-tilmanocept. The anal side of the image is at the top of each panel. (a) Brightfield imaging showing sentinel lymph nodes (white oval) along the right internal iliac artery (white arrowhead) and right external iliac artery (white arrowhead) were visualized using a near-infrared camera. (**b**) standard overlay image. (**c**) *Sensitive* mode image, and (**d**) brightfield image of exposed internal iliac artery bifurcation (black arrowhead) and external iliac artery bifurcation (black arrowhead); the sentinel lymph node (SLN) has been excised. Note overlayed black lines demarcating the right internal and right external arteries in panels (**a**) and (**d**).

Surgery was performed using the *DaVinci Xi Firefly* camera system with preoperative PET/CT imaging as a guide. Using a Firefly camera, lymph node was detected both standard mode (Fig. 3b) and sensitive mode (Fig. 3c). The sensitive mode showed the lymph node more clearly (Fig. 3c). Although the lymph node was difficult to recognize under bright field imaging, fluorescence guidance clearly revealed the tilmanocept-positive lymph node, demonstrating the feasibility and utility of the multimodal tilmanocept navigation for accurate sentinel lymph node identification.

The mean number of resected lymph nodes per animal was 24.25 ± 5.68, with 2.5 ± 1.29 SLNs following the 10% rule. All PET/CT-positive regional lymph nodes were excised. The *Firefly* system identified nine of the 10 PET/CT-positive lymph nodes. The one lymph node that could not be detected via the intra-operative camera was the pudendal artery regional lymph node of Pig #4, which was outside the abdominal cavity and therefore not within the field-of-view of the *FireFly* camera. This pudendal artery regional lymph node in Pig#4 was removed by open surgery because the space between the sacrum and the iliac bone was too narrow and deep to be approached by the *DaVinci Xi*.

Figure 3 displays the representative *Firefly* system images of Pig#3. Figure 3a is a bright field image, the arrow points to a sentinel lymph node between the right internal and the right external iliac arteries; overlayed black lines demarcate the right internal and right external arteries, which are visualized without surgical exposure. The *Firefly* mode (Fig. 3b) revealed fluorescence of the tilmanocept-positive lymph node, which was more clearly displayed by the *sensitive* mode (Fig. 3c). Figure 3d is a brightfield image after excision of the lateral lymph node and surgical exposure of the internal iliac bifurcation (white arrowhead) and external iliac bifurcation (black arrowhead). Black lines demarcate the right internal and right external arteries in panel 3d.

### Ex vivo fluorescence imaging

**Fig. 4:**
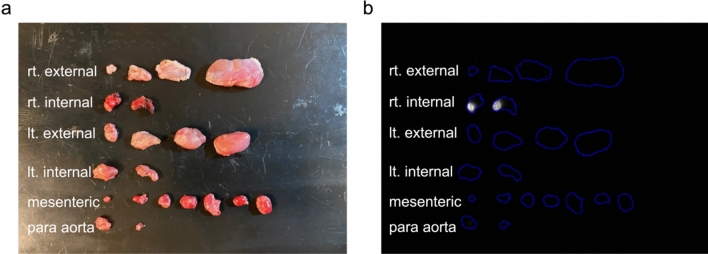
Ex vivo fluorescence imaging of Pig#3 with a hand-held camera were acquired for 10 ms exposure time. Dissected specimens of each lymph node from each regional lymph node: (**a**) bright field, (**b**) optical image demonstrated intra-nodal fluorescence within the right internal lymph nodes.

All SLNs identified by the *Firefly* system were also detectable using *Fluobeam800* at an exposure time of 10 ms (Table 1). Figure 4 is the brightfield (Fig. 4a) and fluorescence images (Fig. 4b) from Pig #3. The optical imager demonstrated intra-nodal fluorescence within two right internal lymph nodes. These two lymph nodes were consistent with the SLNs identified on pre-operative imaging and ex vivo assay of technetium-99m radioactivity.

### Ex vivo radioactivity counting

**Fig. 5:**
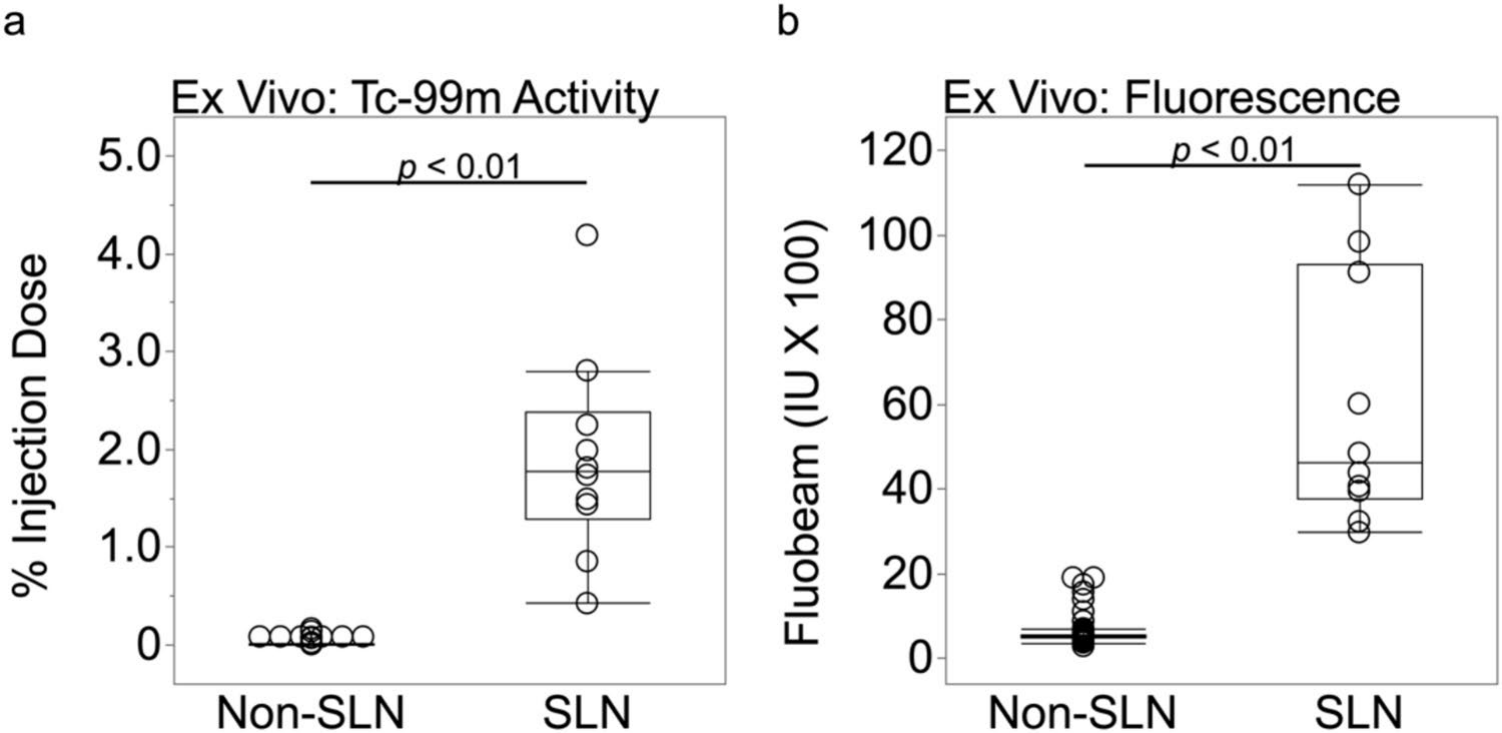
Tilmanocept accumulation by sentinel lymph node was significantly (*P* < 0.01) higher (mean ± SD) than non-sentinel lymph nodes (mean ± SD): (**a**) percent-of-injection dose (%ID), (**b**) maximum fluorescent intensity (IU).

The radioactive activity results for Tc-99m indicated that the mean %ID ± SD for the ten sentinel lymph nodes was 1.90 ± 1.05, which was significantly (*P* < 0.01) different than mean value (0.01 ± 0.02) of the non-sentinel lymph nodes (Fig. 5a). The maximum fluorescent intensity for SLNs and NSLNs were 5963.2 ± 2985.0 and 553.9 ± 199.2, respectively, indicating a statistically significant difference (*P* < 0.01) (Fig. 5b).

## Discussion

In this study, we successfully demonstrated the ability of multimodal tilmanocept to preoperatively image and intra-operatively map sentinel lymph nodes. By combining preoperative PET/CT imaging with intraoperative fluorescent navigation, we demonstrated a significant potential for improving patient selection for LPLND. Avid binding to CD206 allows tilmanocept to remain detectable in lymph nodes for at least 45 h post-injection, enabling precise SLN identification with the intra-operative *Firefly* camera system. The goal of this innovative approach is to reduce unnecessary LPLND procedures, thereby decreasing operative time and minimizing the risk of complications.

In the lower rectum, the lymphatic flow becomes more complex, as it approaches the anus. There are two major lymphatic pathways: the upstream and the lateral direction. Upstream lymphatic flow runs along the superior rectal artery and inferior mesenteric artery within the mesorectum. The lateral lymphatic flow, on the other hand, drains into the basin of lateral pelvic lymph nodes, particularly around the internal iliac artery and obturator artery. This lateral lymphatic flow is responsible for lateral pelvic lymph node metastasis. Currently, there are no clear imaging diagnostic criteria for lymph nodes suspected of metastasis^35^, leading to a lack of established treatment guidelines. Patients with enlarged or borderline-metastatic lateral pelvic lymph nodes are typically treated with a combination of CRT and/or LPLND. However, some patients who undergo LPLND are over-treated^6,9^. Therefore, it is crucial to stratify the patients effectively to ensure that only those who truly need LPLND receive it, especially after CRT, thereby avoiding unnecessary invasive treatments.

SLN biopsy is one of the approaches for stratification. The usefulness of ICG-guided sentinel node biopsy in assessing the metastatic status in lateral pelvic lymph nodes has been reported^20,36^. In these reports, sentinel lymph nodes without metastasis in the lateral pelvic region indicate the absence of metastasis in other non-sentinel pelvic lymph nodes. Moreover, evaluating sentinel lymph nodes may contribute to the detection of metastatic lymph nodes, including micrometastasis^37,38^. However, it should be noted that widespread lymph node metastasis or replacement with cancer cells in lymph nodes inhibits staining with blue dye or ICG^39,40^. Based on the above, patients without obvious lymph node metastasis or metastatic border line lymph nodes, such as 5–10 mm or less, will be considered as suitable candidates for SLN mapping.

The advantage of tilmanocept lies in its versatile labeling capabilities. It can be labeled with gallium-68 for PET/CT imaging or technetium-99m for SPECT/CT imaging. In addition, tilmanocept can also be conjugated with a fluorescent dye such as *IRDye800CW,* enabling near-infrared imaging during surgery^21^ as well as preoperative imaging. Preoperative lymph node mapping provides anatomical information and potentially detects more SLNs^41^. This permits preoperative planning of the surgery and reduction of the time required for intra-operative SLNs mappings^41^.

This fluorescence navigation is effective only for superficial areas up to a depth of 10 mm, with limited fluorescent penetration^42^. Identifying the fluorescence of lymph nodes located deeper than 10 mm, such as those in the lateral pelvic lymph node and mesorectum, remains challenging. In human rectal cancer, lateral pelvic lymph node metastases are located more frequently in the iliac artery region and obturator artery region^43^. These regions are sites deeply embedded within the pelvic cavity with thick fat, and can only be detected via external imaging, such as PET/CT or SPECT/CT.

A specific example of how the preoperative merged PET/CT cross-sections were used to provide 3-dimensional guidance to the surgeon during intraoperative sentinel lymph node mapping are provided by Figs. 1 and 3. The PET/CT-positive lymph node in Fig. 1c was assumed to be located between the right external iliac artery and the internal iliac artery near the bifurcation of the internal iliac arteries (Fig. 3a). The right external iliac artery was traced cranially as a surgical landmark to confirm its anatomical relationship with the right internal iliac artery. Based on preoperative imaging, the lymph node shown in Fig. 1c, located between these arteries, was intraoperatively identified around the expected location using the fluorescent camera. Since the lymph node depicted in Fig. 1b was expected to be located 5.7 mm caudodorsally, lateral to the internal iliac artery from Fig. 1c, dissection was extended in that direction in Fig. 1b. Consequently, during surgery, tracing the right external iliac artery cranially and allowed the surgeon to estimate the approximately location of the target lymph node in Fig. 1 between the right internal and external iliac arteries.

Figure 3d was obtained to confirm the vascular course demonstrated in Fig. 3a, specifically the bifurcation of the internal iliac artery (white arrowhead) and the external iliac artery (black arrowhead). As indicated by the black lines in Fig. 3a, these arterial structures can be easily visualized without extensive surgical dissection. Therefore, they serve as useful anatomical landmarks during the procedure. Consequently, with the aid of fluorescence imaging, the sentinel lymph nodes could be identified without the need for complete vascular exposure as shown in Fig. 3d.

The fluorescence of lateral pelvic sentinel lymph nodes in deep region may not be visible from the dissection surface after TME. In such cases, fluorescence should be detected by dissecting through the fat, but vessels, arteries, and the ureter duct are contained in this fat. Random excision to detect fluorescence may cause morbidity and take more time. If there is preoperative lymph node site information, the sentinel node can be accessed safely and accurately using anatomical landmarks and lymph node mapping. If pre-operative SPECT/CT imaging does not detect a SLN, unnecessary dissection maneuvers can be omitted, which may lead to a lower incidence of complications related to LPLND.

One example of the benefit of preoperative lymph node mapping was seen in a pudendal artery regional lymph node in case 4. All SLNs except the pudendal artery regional lymph node could be detected by fluorescence due to less fat. However, the pudendal artery regional SLN existed in the distal pudendal artery region between the sacral and iliac bone spaces. The *Da Vinci* camera was unable to identify fluorescence due to being physically inaccessible. This lymph node is considered a site of systemic metastasis and is not typically resected in clinical practice. In this preclinical study, the purpose was to confirm the consistency between preoperative PET-CT images and intra- and post-operative fluorescent images. The surgery was converted to an open approach the preoperative PET-CT images showed that the sentinel lymph node was outside the detection range of the *Firefly* camera. The sentinel status of the target lymph node was confirmed intraoperatively by the fluorescent imaging and by Tc-99m radioactivity measurement. This complementary strategy, preoperative PET/CT mapping followed by intraoperative fluorescence navigation, demonstrates that even deeply situated SLNs can be accurately and efficiently identified and removed.

Tilmanocept can reside in lymph nodes for up to 72 h after injection^29^; this study demonstrated a fluorescent signal within sentinel lymph nodes at 45 h post-administration. The extended retention of tilmanocept in sentinel lymph nodes allows surgeries to be performed the day after imaging, significantly reducing radiation exposure to the staff, and ensuring a bright fluorescent emission for detection by the *Firefly* camera.

We envision the following implementation of a technetium and fluorescent version of tilmanocept for sentinel lymph node mapping of rectal cancer. The bi-modal tilmanocept would be administered within the submucosa of the rectal wall near the tumor. One hour later, a 20-min SPECT/CT of the subject’s pelvis would be acquired. A radiologist would examine the resulting attenuation-corrected cross-sectional images and identify sentinel lymph nodes based on SUVs and the 10%-rule. After imaging, the operation could be performed up to 72 h after administration of the fluorescent-tilmanocept. Prior to excision, fluorescent-tilmanocept within each sentinel lymph node would be visualized by the fluorescence imaging subsystem of the surgical robot. Each fluorescent lateral pelvic lymph node packet would be submitted for histologic examinate as a sentinel lymph node.

Based on recent Phase I and II clinical trials of IRDye800CW-conjugated antibodies, we do not expect any toxicity issues with IRDye800CW-conjugated tilmanocept. There are two reasons for this expectation. First, is the safety record of tilmanocept. Since its FDA approval^44^ in 2013 for sentinel lymph node mapping, Tc-99m-tilmanocept (Tradename: Lymphoseek) has maintained an excellent safety record; it has never had a serious adverse event and extremely infrequent non-serious adverse events. The molar dose used for Tc-99m-tilmanocept sentinel lymph node mapping studies^45^, 3.0 nmol, is similar to the amount used in this study, 1.5 nmol. Given the high detection sensitivity of the *Firefly* camera exhibited in these studies, we expect the 1.5 nmol dose to be more than satisfactory for human clinical studies. Second, of the many clinical trials conducted with IRDye800CW-conjugated antibodies, none have reported any adverse events beyond toxicities associated with the targeting antibodies. A phase I/II clinical trial of IRDye800CW-nimotuzumab^46^ in patients with resectable non-small cell lung cancer concluded that IRDye800CW conjugated to nimotuzumab was safe and did not exhibit toxicities commonly associated with EGFR targeting antibodies^47^. The study used a 30-min i.v. administration of either 25 or 100 mg (666 nmol) of antibody conjugated with an average of 1.2 dyes per antibody. A second study of IRDye800CW-panitumumab in patients with head and neck squamous cell carcinoma^48^ reported only mild, infusion-related symptoms with no serious adverse events attributable to the fluorescent-antibody. The study administrated either a 50- or 100-mg (666 nmol) dose of antibody conjugated with a 1.5 dyes per antibody. If an IRDye800CW-tilmanocept clinical trial was conducted using the same molar dose (1.5 nmol) and the same average number dyes per tilmanocept (1.5) as employed in this study, the amount of dye used in the IRDye800CW-tilmanocept phase I study would be 444-fold less dye than the IRDye800CW-antibody trials. A standard GLP biosafety study, which will be conducted prior to an IRDye800CW-tilmanocept Phase I trial, typically employs a scaled dose that is 500-fold greater than the clinical trial dose^49^. Consequently, the IRDye800CW-tilmanocept biosafety study will employ a test dose 200,000-fold higher that the dose used in the IRDye800CW-antibody trials.

The strengths of this study are (1) the use of an animal model to demonstrate the rapid and sustained accumulation of radiolabeled-fluorescent-tilmanocept, (2) a demonstration of the potential significance of pre-operative detection of sentinel lymph nodes invisible field of the surgical robot, and (3) a demonstration of fluorescent detection of bi-modal tilmanocept by a standard robotic surgical system. The limitation of this study is that it is an examination of a limited number of pigs. We only injected tilmanocept into the submucosa of the lateral wall just above the dentate line, not the anterior and posterior sides and proximal rectum. In addition, there may be differences in lymphatic flow between humans and pigs due to different anatomy.

## Conclusion

This study demonstrated the ability of pre-operative imaging and intra-operative detection of radiolabeled fluorescent-tilmanocept to efficiently identify sentinel lymph nodes. Sentinel lymph node identification of lateral pelvic lymph nodes using dual-labeled tilmanocept may provide an alternative strategy to the dissection of lateral lymph nodes for all tumors below the peritoneal reflection. PET/CT or SPECT/CT pre-operative sentinel lymph node imaging can potentially reduce morbidity and surgical time by identifying patients who will not require lateral pelvic lymph node dissections.

## Data Availability

The data that support the findings of this study are available from the corresponding author, RO, upon reasonable request.
